# Crystal structure of 2-hy­droxy-2-(2-oxo­cyclo­hept­yl)-2,3-di­hydro-1*H*-indene-1,3-dione

**DOI:** 10.1107/S2056989015016126

**Published:** 2015-09-12

**Authors:** P. Kaleel Ahamed, N. Srinivasan, R. Ranjith Kumar, R. V. Krishnakumar

**Affiliations:** a Department of Physics, Dr. Zakir Husain College, Ilayankudi, Sivagangai District 625 009, India; b Department of Physics, Thiagarajar College, Madurai 625 009, India; c School of Chemistry, Madurai Kamaraj University, Madurai 625 021, India

**Keywords:** crystal structure, indene-1,3-dione, hydrogen bonding, π–π stacking

## Abstract

In the title compound, C_16_H_16_O_4_, the five-membered ring of the indene-1,3-dione unit adopts a twist conformation, whereas the seven-membered ring adopts a twist–chair conformation. In the crystal, mol­ecules are linked by O—H⋯O hydrogen bonds, weak C—H⋯O hydrogen bonds and π–π stacking [centroid-to-centroid distance = 3.7373 (8) Å] into a three-dimensional supra­molecular architecture.

## Related literature   

For the background and potential applications of the title compound, see: Andreu *et al.* (2009 ▸); Fun *et al.* (2009 ▸); Ghalib *et al.* (2011 ▸); Uk Kim *et al.* (2004 ▸); Penthala *et al.* (2009 ▸); Sundar *et al.* (2010 ▸); Yao *et al.* (2006*a* ▸,*b* ▸).
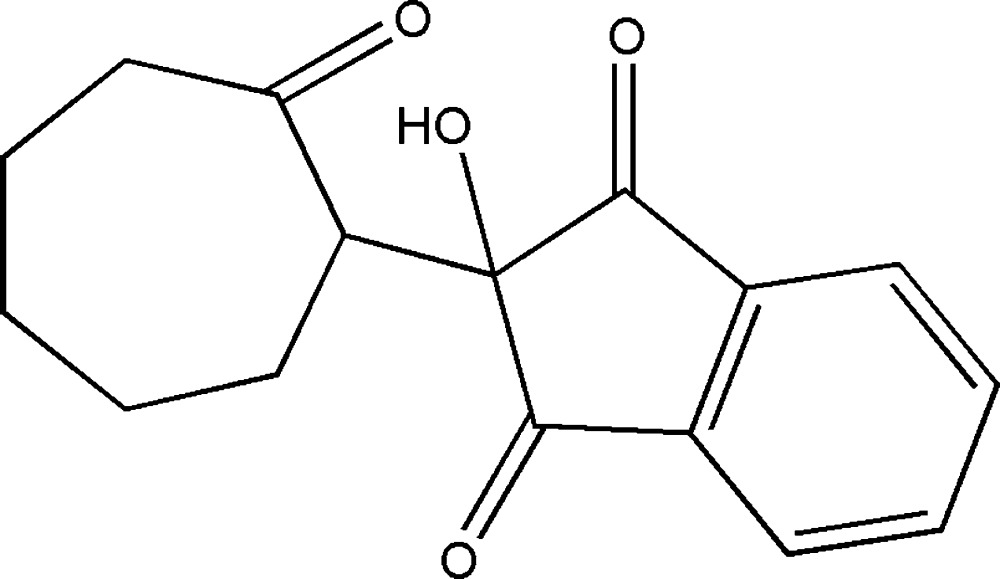


## Experimental   

### Crystal data  

C_16_H_16_O_4_
*M* *_r_* = 272.29
Orthorhombic, 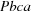

*a* = 7.4131 (5) Å
*b* = 18.8596 (13) Å
*c* = 19.0166 (13) Å
*V* = 2658.7 (3) Å^3^
*Z* = 8
Mo *K*α radiation
μ = 0.10 mm^−1^
*T* = 294 K
0.30 × 0.23 × 0.18 mm

### Data collection  

Bruker SMART APEXII CCD diffractometer
Absorption correction: multi-scan (*SADABS*; Bruker, 2009 ▸) *T*_min_ = 0.978, *T*_max_ = 0.986
28734 measured reflections
3191 independent reflections
2849 reflections with *I* > 2σ(*I*)
*R*_int_ = 0.020

### Refinement  

*R*[*F* ^2^ > 2σ(*F* ^2^)] = 0.045
*wR*(*F* ^2^) = 0.119
*S* = 1.03
3191 reflections
185 parameters
H atoms treated by a mixture of independent and constrained refinement
Δρ_max_ = 0.33 e Å^−3^
Δρ_min_ = −0.20 e Å^−3^

### 

Data collection: *APEX2* (Bruker, 2009 ▸); cell refinement: *SAINT* (Bruker, 2009 ▸); data reduction: *SAINT*; program(s) used to solve structure: *SHELXS2013* (Sheldrick, 2008 ▸); program(s) used to refine structure: *SHELXL2014* (Sheldrick, 2015 ▸); molecular graphics: *PLATON* (Spek, 2009 ▸); software used to prepare material for publication: *publCIF* (Westrip, 2010 ▸).

## Supplementary Material

Crystal structure: contains datablock(s) I, global. DOI: 10.1107/S2056989015016126/xu5867sup1.cif

Structure factors: contains datablock(s) I. DOI: 10.1107/S2056989015016126/xu5867Isup2.hkl

Supporting information file. DOI: 10.1107/S2056989015016126/xu5867Isup3.cdx

Supporting information file. DOI: 10.1107/S2056989015016126/xu5867Isup4.cml

**Fig. 1.**
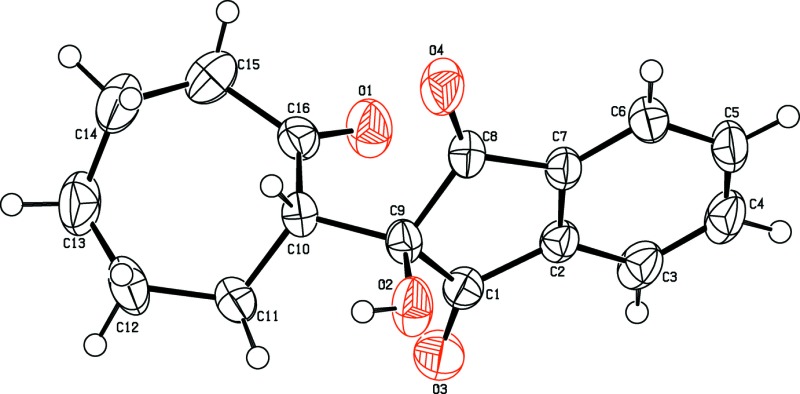
The molecular structure of (I), with atom labels and 50% probability displacement ellipsoids for non-H atoms.

**Fig. 2.**
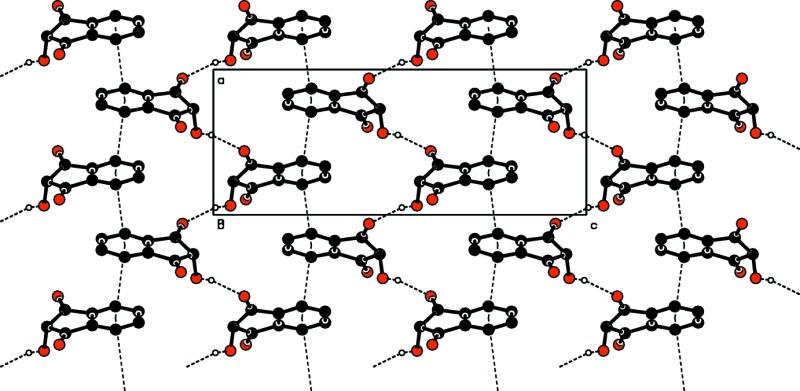
A view of the molecular aggregation down the a axis. Ring systems and H atoms that are not involved in hydrogen bonding have been omitted for clarity.

. DOI: 10.1107/S2056989015016126/xu5867fig1.tif
The mol­ecular structure of (I), with atom labels and 50% probability displacement ellipsoids for non-H atoms.

a . DOI: 10.1107/S2056989015016126/xu5867fig2.tif
A view of the mol­ecular aggregation down the *a* axis. Ring systems and H atoms that are not involved in hydrogen bonding have been omitted for clarity.

CCDC reference: 1421141

Additional supporting information:  crystallographic information; 3D view; checkCIF report

## Figures and Tables

**Table 1:** Hydrogen-bond geometry (, )

*D*H*A*	*D*H	H*A*	*D* *A*	*D*H*A*
O2H2O4^i^	0.80(2)	1.99(2)	2.7707(13)	163(2)
C4H4O1^ii^	0.93	2.48	3.2758(17)	144
C15H15*B*O3^iii^	0.97	2.47	3.3998(19)	160

Symmetry codes: (i) 
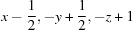
; (ii) 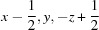; (iii) 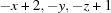.

## supplementary crystallographic information

### S1. Comment

In the continuation of studies of ninhydrin reactions *viz*. 2-acetonyl-2-hydroxyindan-1,3-dione (Fun *et al.*, 2009), rac-2-(2-amino-4-pxp-4,5-dihydro-1,3-thiazol-5-yl)-2- hydroxyindane-1,3-dione (Penthala *et al.*, 2009), rac-2-hydroxy-2-(2-oxocyclopentyl)-1*H*-indene- 1,3(2*H*)-dione (Sundar *et al.*, 2010), 2-hydroxy-2- (3-oxobutan-2-yl)indan-1,3-dione, we have undertaken the structural analysis of the title compound, the indene-1,3(2*H*)-dione moiety belongs to an important class of luminescent materials which is used as a strong electron acceptor in organic light-emitting diodes (Yao *et al.*, 2006a,b; Andreu *et al.*, 2009; Kim *et al.*, 2004). The derivatives of indandione is a promising materials in the field of photonics. It is also used in the first stage of forensic identification of latent fingerprints.

The measure of angle strain is 5.65° which is comparable with calculated crystal structure data value of 6.5°.

The title compound 2-hydroxy-2-(2-oxocycloheptyl)-2,3-dihydro-1*H*-indene-1,3-dione crystallizes in orthorhombic space group Pbca. The five-membered ring adopts twist conformation on C8—C9 with Q = 0.1502 Å and φ = 302.24°. In the crystal structure, molecules are linked by intermolecular O—H···O and C—H···O hydrogen bonds. The symmetry related six-membered spiro rings show π-π interactions with distance of 3.7373 (8) Å (Fig. 2).

The O—H···O hydrogen bonding form a infinite linear hydrogen bonding chain C(11) extending along *a* axis.

The mean plane of oxocycloheptyl and fused ring of indene make an angle of 61.945 °. The substituent oxygen O1 deviates from the mean plane of oxocycloheptyl ring by -0.9121 Å

### S2. Experimental

A mixture of cycloheptanone (1 mmol) and ninhydrin (1 mmol) was taken in a boiling tube and was subjected to microwave irradiation for 5 minutes. The progress of reaction was monitored by thin layer chromatography after each one minute of irradiation. After completion of reaction as evident from TLC, the residue was purified by column chromatography by using petroleum ether and ethyl acetate 65:35 *v*/*v* mixture as an eluent to afford the product. The product was recrystallized from ethyl acetate.

### S3. Refinement

H atoms were positioned geometrically and refined using a riding model with C—H = 0.95–0.99 Å and with *U*_iso_(H) = 1.2 (1.5 for methyl groups) times *U*_eq_(C).

### Crystal data

**Table:**

C_16_H_16_O_4_	*D*_x_ = 1.361 Mg m^−^^3^*D*_m_ = 1.35 Mg m^−^^3^*D*_m_ measured by floatation method
*M**_r_* = 272.29	Mo *K*α radiation, λ = 0.71073 Å
Orthorhombic, *P**b**c**a*	Cell parameters from 7469 reflections
*a* = 7.4131 (5) Å	θ = 2.4–27.8°
*b* = 18.8596 (13) Å	µ = 0.10 mm^−^^1^
*c* = 19.0166 (13) Å	*T* = 294 K
*V* = 2658.7 (3) Å^3^	Needle, colourless
*Z* = 8	0.30 × 0.23 × 0.18 mm
*F*(000) = 1152	

### Data collection

**Table:**

Bruker SMART APEXII CCD diffractometer	2849 reflections with *I* > 2σ(*I*)
Radiation source: fine-focus sealed tube	*R*_int_ = 0.020
φ and ω scans	θ_max_ = 28.0°, θ_min_ = 2.2°
Absorption correction: multi-scan (*SADABS*; Bruker, 2009)	*h* = −9→9
*T*_min_ = 0.978, *T*_max_ = 0.986	*k* = −24→24
28734 measured reflections	*l* = −24→25
3191 independent reflections	

### Refinement

**Table:**

Refinement on *F*^2^	0 restraints
Least-squares matrix: full	Hydrogen site location: inferred from neighbouring sites
*R*[*F*^2^ > 2σ(*F*^2^)] = 0.045	H atoms treated by a mixture of independent and constrained refinement
*wR*(*F*^2^) = 0.119	*w* = 1/[σ^2^(*F*_o_^2^) + (0.0599*P*)^2^ + 0.7812*P*] where *P* = (*F*_o_^2^ + 2*F*_c_^2^)/3
*S* = 1.03	(Δ/σ)_max_ < 0.001
3191 reflections	Δρ_max_ = 0.33 e Å^−^^3^
185 parameters	Δρ_min_ = −0.20 e Å^−^^3^

### Special details

**Table:**

Geometry. All e.s.d.'s (except the e.s.d. in the dihedral angle between two l.s. planes) are estimated using the full covariance matrix. The cell e.s.d.'s are taken into account individually in the estimation of e.s.d.'s in distances, angles and torsion angles; correlations between e.s.d.'s in cell parameters are only used when they are defined by crystal symmetry. An approximate (isotropic) treatment of cell e.s.d.'s is used for estimating e.s.d.'s involving l.s. planes.

### Fractional atomic coordinates and isotropic or equivalent isotropic displacement parameters (Å^2^)

**Table:**

	*x*	*y*	*z*	*U*_iso_*/*U*_eq_	
O1	1.03172 (15)	0.08184 (7)	0.44453 (5)	0.0593 (3)	
O2	0.56285 (14)	0.19613 (6)	0.45415 (5)	0.0525 (3)	
O3	0.60520 (16)	0.04223 (5)	0.41294 (5)	0.0565 (3)	
O4	0.93803 (17)	0.25552 (5)	0.41716 (5)	0.0570 (3)	
C1	0.68387 (16)	0.09552 (6)	0.39677 (6)	0.0357 (3)	
C2	0.73825 (16)	0.11650 (6)	0.32474 (6)	0.0343 (3)	
C3	0.70883 (18)	0.08049 (8)	0.26203 (7)	0.0446 (3)	
H3	0.6582	0.0354	0.2617	0.053*	
C4	0.75714 (19)	0.11383 (9)	0.20020 (7)	0.0509 (4)	
H4	0.7383	0.0908	0.1576	0.061*	
C5	0.83328 (19)	0.18099 (9)	0.20053 (6)	0.0487 (3)	
H5	0.8608	0.2028	0.1580	0.058*	
C6	0.86906 (18)	0.21611 (7)	0.26286 (6)	0.0427 (3)	
H6	0.9238	0.2605	0.2631	0.051*	
C7	0.82010 (16)	0.18254 (6)	0.32509 (6)	0.0335 (2)	
C8	0.84477 (17)	0.20639 (6)	0.39815 (6)	0.0355 (3)	
C9	0.72848 (16)	0.15854 (6)	0.44602 (6)	0.0334 (2)	
C10	0.81939 (16)	0.14094 (6)	0.51591 (6)	0.0343 (2)	
H10	0.8396	0.1855	0.5412	0.041*	
C11	0.70788 (19)	0.09172 (7)	0.56410 (7)	0.0434 (3)	
H11A	0.5807	0.1009	0.5563	0.052*	
H11B	0.7312	0.0429	0.5509	0.052*	
C12	0.7486 (3)	0.10069 (9)	0.64220 (7)	0.0589 (4)	
H12A	0.7260	0.1497	0.6549	0.071*	
H12B	0.6645	0.0716	0.6686	0.071*	
C13	0.9388 (3)	0.08166 (10)	0.66515 (8)	0.0679 (5)	
H13A	0.9607	0.0323	0.6534	0.081*	
H13B	0.9459	0.0860	0.7159	0.081*	
C14	1.0880 (2)	0.12630 (9)	0.63284 (8)	0.0594 (4)	
H14A	1.1938	0.1232	0.6627	0.071*	
H14B	1.0494	0.1754	0.6324	0.071*	
C15	1.1415 (2)	0.10527 (8)	0.55851 (8)	0.0547 (4)	
H15A	1.2401	0.1358	0.5441	0.066*	
H15B	1.1886	0.0573	0.5604	0.066*	
C16	1.00134 (17)	0.10759 (6)	0.50196 (7)	0.0380 (3)	
H2	0.549 (3)	0.2103 (10)	0.4936 (11)	0.070 (6)*	

### Atomic displacement parameters (Å^2^)

**Table:**

	*U*^11^	*U*^22^	*U*^33^	*U*^12^	*U*^13^	*U*^23^
O1	0.0519 (6)	0.0860 (8)	0.0399 (5)	0.0216 (5)	0.0051 (4)	−0.0029 (5)
O2	0.0512 (6)	0.0737 (7)	0.0326 (5)	0.0284 (5)	0.0016 (4)	−0.0022 (4)
O3	0.0654 (7)	0.0510 (6)	0.0530 (6)	−0.0195 (5)	−0.0038 (5)	0.0066 (4)
O4	0.0843 (8)	0.0484 (5)	0.0381 (5)	−0.0224 (5)	−0.0097 (5)	−0.0021 (4)
C1	0.0347 (6)	0.0388 (6)	0.0335 (6)	0.0007 (4)	−0.0020 (4)	−0.0002 (4)
C2	0.0325 (5)	0.0405 (6)	0.0298 (5)	0.0033 (4)	−0.0018 (4)	−0.0050 (4)
C3	0.0420 (7)	0.0523 (7)	0.0394 (7)	0.0029 (5)	−0.0053 (5)	−0.0148 (5)
C4	0.0451 (7)	0.0768 (10)	0.0308 (6)	0.0108 (7)	−0.0049 (5)	−0.0160 (6)
C5	0.0429 (7)	0.0771 (9)	0.0261 (6)	0.0113 (6)	0.0019 (5)	0.0035 (6)
C6	0.0418 (6)	0.0535 (7)	0.0327 (6)	0.0011 (5)	0.0009 (5)	0.0058 (5)
C7	0.0340 (5)	0.0403 (6)	0.0261 (5)	0.0030 (4)	−0.0009 (4)	−0.0013 (4)
C8	0.0452 (6)	0.0337 (5)	0.0277 (5)	0.0015 (5)	−0.0028 (4)	−0.0005 (4)
C9	0.0367 (6)	0.0376 (5)	0.0258 (5)	0.0057 (4)	0.0017 (4)	−0.0003 (4)
C10	0.0399 (6)	0.0372 (6)	0.0257 (5)	0.0024 (5)	0.0003 (4)	0.0015 (4)
C11	0.0440 (7)	0.0514 (7)	0.0348 (6)	−0.0021 (5)	0.0044 (5)	0.0083 (5)
C12	0.0780 (11)	0.0668 (9)	0.0319 (6)	−0.0066 (8)	0.0087 (7)	0.0102 (6)
C13	0.0937 (13)	0.0668 (10)	0.0431 (8)	−0.0138 (9)	−0.0193 (8)	0.0195 (7)
C14	0.0743 (10)	0.0544 (8)	0.0494 (8)	−0.0069 (7)	−0.0244 (7)	0.0063 (6)
C15	0.0475 (8)	0.0568 (8)	0.0597 (9)	0.0069 (6)	−0.0157 (7)	−0.0008 (7)
C16	0.0379 (6)	0.0400 (6)	0.0360 (6)	0.0004 (5)	0.0017 (5)	0.0066 (5)

### Geometric parameters (Å, º)

**Table:**

O1—C16	1.2163 (16)	C9—C10	1.5267 (15)
O2—C9	1.4262 (14)	C10—C16	1.5118 (17)
O2—H2	0.80 (2)	C10—C11	1.5442 (16)
O3—C1	1.2018 (15)	C10—H10	0.9800
O4—C8	1.2113 (15)	C11—C12	1.5249 (18)
C1—C2	1.4818 (16)	C11—H11A	0.9700
C1—C9	1.5489 (16)	C11—H11B	0.9700
C2—C7	1.3856 (17)	C12—C13	1.519 (3)
C2—C3	1.3894 (16)	C12—H12A	0.9700
C3—C4	1.381 (2)	C12—H12B	0.9700
C3—H3	0.9300	C13—C14	1.520 (3)
C4—C5	1.387 (2)	C13—H13A	0.9700
C4—H4	0.9300	C13—H13B	0.9700
C5—C6	1.3833 (18)	C14—C15	1.521 (2)
C5—H5	0.9300	C14—H14A	0.9700
C6—C7	1.3904 (16)	C14—H14B	0.9700
C6—H6	0.9300	C15—C16	1.4961 (19)
C7—C8	1.4718 (15)	C15—H15A	0.9700
C8—C9	1.5448 (16)	C15—H15B	0.9700
			
C9—O2—H2	112.0 (14)	C9—C10—H10	107.9
O3—C1—C2	126.28 (11)	C11—C10—H10	107.9
O3—C1—C9	126.20 (11)	C12—C11—C10	113.91 (12)
C2—C1—C9	107.22 (9)	C12—C11—H11A	108.8
C7—C2—C3	120.82 (11)	C10—C11—H11A	108.8
C7—C2—C1	110.77 (9)	C12—C11—H11B	108.8
C3—C2—C1	128.33 (12)	C10—C11—H11B	108.8
C4—C3—C2	117.88 (13)	H11A—C11—H11B	107.7
C4—C3—H3	121.1	C13—C12—C11	115.93 (14)
C2—C3—H3	121.1	C13—C12—H12A	108.3
C3—C4—C5	121.17 (12)	C11—C12—H12A	108.3
C3—C4—H4	119.4	C13—C12—H12B	108.3
C5—C4—H4	119.4	C11—C12—H12B	108.3
C6—C5—C4	121.29 (12)	H12A—C12—H12B	107.4
C6—C5—H5	119.4	C12—C13—C14	115.37 (13)
C4—C5—H5	119.4	C12—C13—H13A	108.4
C5—C6—C7	117.47 (13)	C14—C13—H13A	108.4
C5—C6—H6	121.3	C12—C13—H13B	108.4
C7—C6—H6	121.3	C14—C13—H13B	108.4
C2—C7—C6	121.30 (11)	H13A—C13—H13B	107.5
C2—C7—C8	109.50 (10)	C13—C14—C15	114.90 (14)
C6—C7—C8	129.20 (11)	C13—C14—H14A	108.5
O4—C8—C7	125.90 (11)	C15—C14—H14A	108.5
O4—C8—C9	126.13 (10)	C13—C14—H14B	108.5
C7—C8—C9	107.96 (9)	C15—C14—H14B	108.5
O2—C9—C10	113.19 (9)	H14A—C14—H14B	107.5
O2—C9—C8	104.70 (9)	C16—C15—C14	118.64 (13)
C10—C9—C8	113.18 (10)	C16—C15—H15A	107.7
O2—C9—C1	105.26 (10)	C14—C15—H15A	107.7
C10—C9—C1	116.99 (9)	C16—C15—H15B	107.7
C8—C9—C1	102.19 (9)	C14—C15—H15B	107.7
C16—C10—C9	109.36 (9)	H15A—C15—H15B	107.1
C16—C10—C11	109.37 (9)	O1—C16—C15	120.34 (12)
C9—C10—C11	114.26 (10)	O1—C16—C10	119.27 (11)
C16—C10—H10	107.9	C15—C16—C10	120.38 (11)
			
O3—C1—C2—C7	177.60 (13)	O3—C1—C9—O2	−76.24 (15)
C9—C1—C2—C7	3.64 (13)	C2—C1—C9—O2	97.72 (11)
O3—C1—C2—C3	0.9 (2)	O3—C1—C9—C10	50.41 (17)
C9—C1—C2—C3	−173.10 (12)	C2—C1—C9—C10	−135.63 (10)
C7—C2—C3—C4	−2.52 (19)	O3—C1—C9—C8	174.60 (13)
C1—C2—C3—C4	173.93 (12)	C2—C1—C9—C8	−11.44 (12)
C2—C3—C4—C5	0.3 (2)	O2—C9—C10—C16	−174.64 (10)
C3—C4—C5—C6	2.1 (2)	C8—C9—C10—C16	−55.70 (12)
C4—C5—C6—C7	−2.2 (2)	C1—C9—C10—C16	62.72 (13)
C3—C2—C7—C6	2.51 (18)	O2—C9—C10—C11	62.41 (14)
C1—C2—C7—C6	−174.52 (11)	C8—C9—C10—C11	−178.65 (10)
C3—C2—C7—C8	−176.47 (11)	C1—C9—C10—C11	−60.24 (13)
C1—C2—C7—C8	6.50 (14)	C16—C10—C11—C12	83.76 (14)
C5—C6—C7—C2	−0.13 (18)	C9—C10—C11—C12	−153.30 (12)
C5—C6—C7—C8	178.63 (12)	C10—C11—C12—C13	−63.91 (18)
C2—C7—C8—O4	166.80 (13)	C11—C12—C13—C14	62.5 (2)
C6—C7—C8—O4	−12.1 (2)	C12—C13—C14—C15	−79.9 (2)
C2—C7—C8—C9	−14.11 (13)	C13—C14—C15—C16	59.78 (19)
C6—C7—C8—C9	167.02 (12)	C14—C15—C16—O1	−169.58 (14)
O4—C8—C9—O2	84.79 (15)	C14—C15—C16—C10	9.5 (2)
C7—C8—C9—O2	−94.30 (11)	C9—C10—C16—O1	−19.61 (16)
O4—C8—C9—C10	−38.94 (17)	C11—C10—C16—O1	106.21 (14)
C7—C8—C9—C10	141.98 (10)	C9—C10—C16—C15	161.33 (12)
O4—C8—C9—C1	−165.63 (13)	C11—C10—C16—C15	−72.85 (15)
C7—C8—C9—C1	15.28 (12)		

### Hydrogen-bond geometry (Å, º)

**Table:**

*D*—H···*A*	*D*—H	H···*A*	*D*···*A*	*D*—H···*A*
O2—H2···O4^i^	0.80 (2)	1.99 (2)	2.7707 (13)	163 (2)
C4—H4···O1^ii^	0.93	2.48	3.2758 (17)	144
C15—H15*B*···O3^iii^	0.97	2.47	3.3998 (19)	160

Symmetry codes: (i) *x*−1/2, −*y*+1/2, −*z*+1; (ii) *x*−1/2, *y*, −*z*+1/2; (iii) −*x*+2, −*y*, −*z*+1.
