# Supplementary figures and images for: Crystal structure of 2-hy­droxy-2-(2-oxo­cyclo­hept­yl)-2,3-di­hydro-1H-indene-1,3-dione
Source: Acta Crystallogr E Crystallogr Commun. 2015 Sep 12;71(Pt 10):o715–6. doi: 10.1107/S2056989015016126 (PMC4647374; doi:10.1107/S2056989015016126)

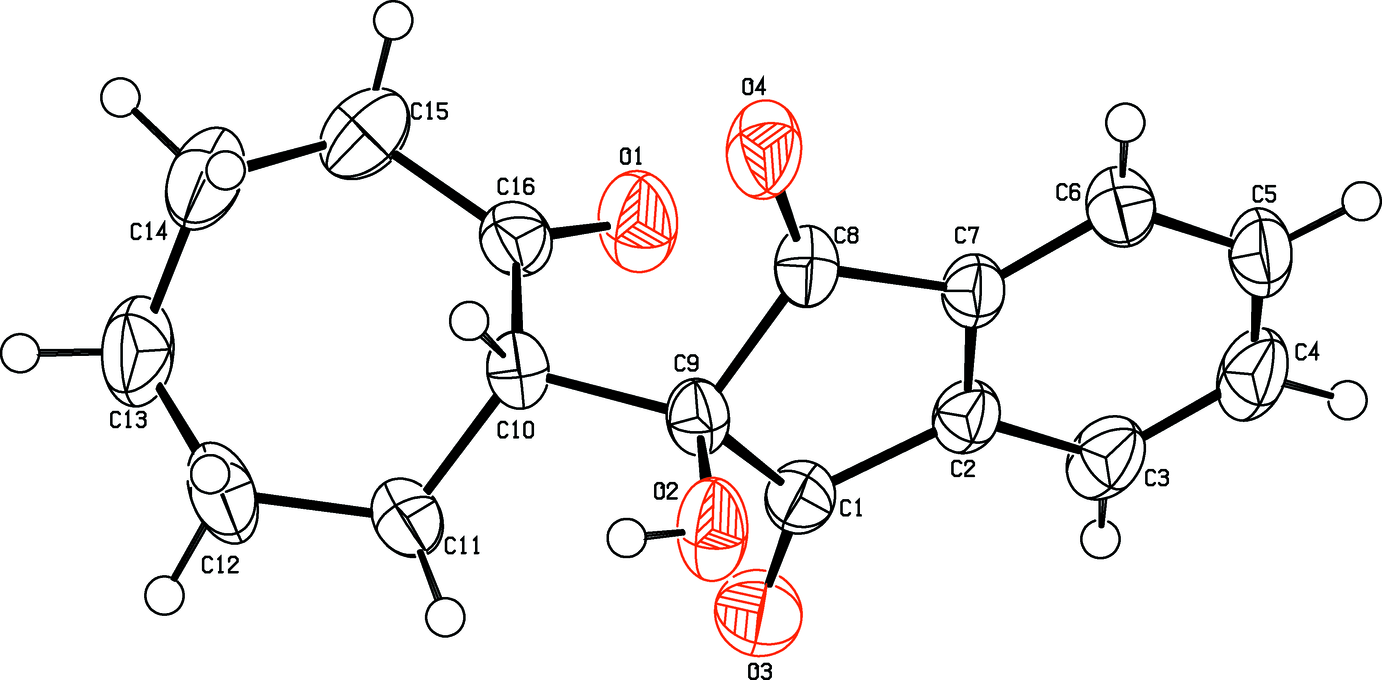

Supplement: Supplementary file 5 [file e-71-0o715-fig1.tif]

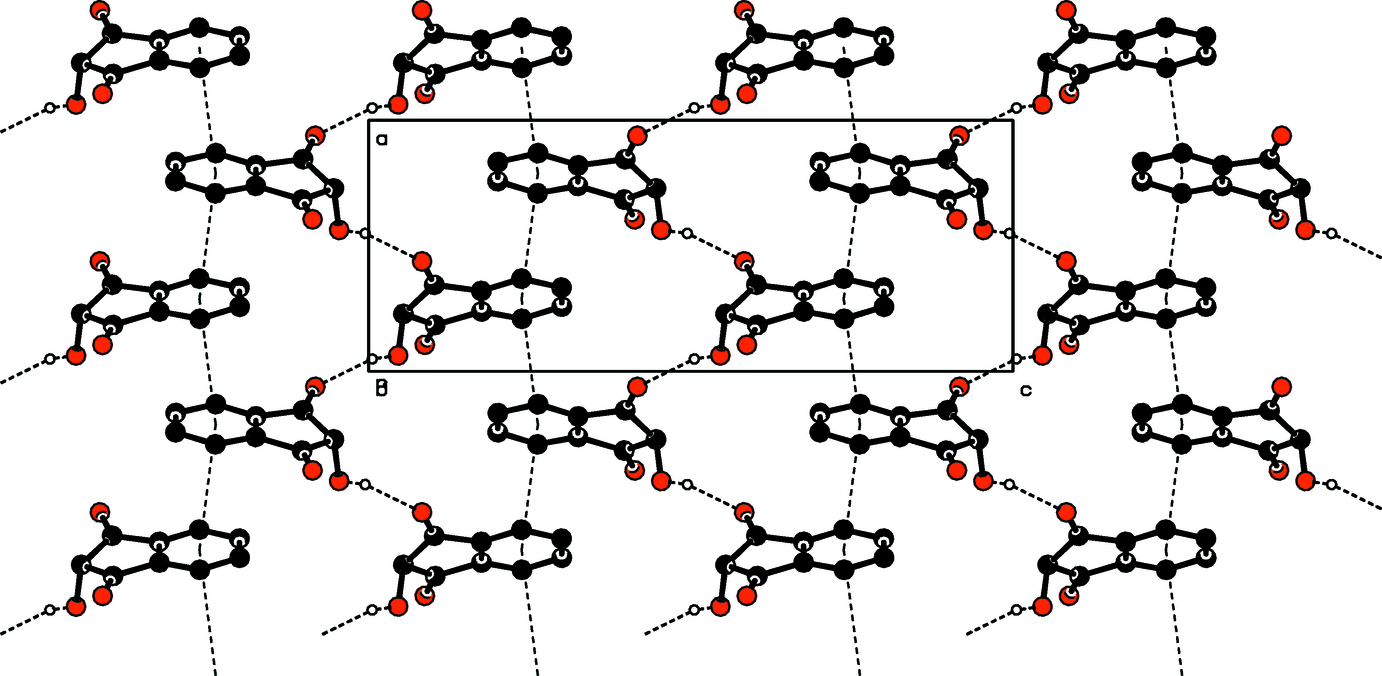

Supplement: Supplementary file 6 [file e-71-0o715-fig2.tif]
